# Treatment of Acquired von Willebrand Disease due to Extracorporeal Membrane Oxygenation in a Pediatric COVID-19 Patient with Vonicog Alfa: A Case Report and Literature Review

**DOI:** 10.1055/a-2008-4367

**Published:** 2023-02-23

**Authors:** Lars Heubner, Karolin Trautmann-Grill, Oliver Tiebel, Martin Mirus, Andreas Güldner, Axel Rand, Peter Markus Spieth

**Affiliations:** 1 Department of Anesthesiology and Intensive Care Medicine, University Hospital Carl Gustav Carus at the Technische Universität Dresden, Dresden, Germany; 2 Department of Internal Medicine I, University Hospital Carl Gustav Carus at the Technische Universität Dresden, Dresden. Germany; 3 Institute of Clinical Chemistry, University Hospital Carl Gustav Carus at the Technische Universität Dresden, Dresden. Germany

**Keywords:** acquired coagulation disorders, von Willebrand disease, extracorporal circulation

## Abstract

Acquired von Willebrand disease (aVWD) is frequently observed in patients with the need for extracorporeal membrane oxygenation (ECMO). aVWD can be treated by plasma-derived concentrates containing factor VIII (FVIII) and/or von Willebrand factor (VWF) and recombinant VWF concentrate as well as adjuvant therapies such as tranexamic acid and desmopressin. However, all of these therapeutic options possibly cause thromboembolism. Therefore, the optimal treatment remains uncertain. This report presents a case of a 16-year-old patient suffering from severe acute respiratory distress syndrome due to coronavirus disease 2019 with the need of ECMO support. Our patient developed aVWD under ECMO therapy characterized by loss of high-molecular-weight multimers (HMWM) and severe bleeding symptoms following endoscopic papillotomy due to sclerosing cholangitis. At the same time standard laboratory parameters showed hypercoagulability with increased fibrinogen level and platelet count. The patient was successfully treated with recombinant VWF concentrate (rVWF; vonicog alfa; Veyvondi) combined with topic tranexamic acid application and cortisone therapy. rVWF concentrate vonicog alfa is characterized by ultra-large multimers and absence of FVIII. Patient could be successfully weaned from ECMO support after 72 days. Multimer analysis 1 week after ECMO decannulation showed an adequate reappearance of HMWM.

## Introduction

Hemorrhagic events are frequently observed in critically ill patients with the need for extracorporeal membrane oxygenation (ECMO) therapy. 1 2 A recent large cohort study investigating 210 ECMO patients suggested, that severe bleeding complication is more frequent in coronavirus disease 2019 (COVID-19) by severe acute respiratory syndrome coronavirus-2 (SARS-CoV-2) than in other viral infections. 3 Contrary to these, COVID-19 is characterized by immunothrombosis and other immunological reactions. 4 5 6 7 8 Due to low incidence, little is known about pediatric COVID-19 ECMO patients. Considering the limited evidence, pediatric patients seem to have a milder clinical course and better prognosis of bleeding complications than adults, 9 but sepsis-induced coagulopathy is also frequently observed in critical ill pediatric patients. 10 Acquired von Willebrand disease (aVWD) is frequently diagnosed in ECMO patients 2 11 and gastrointestinal (GI) bleeding events are common. 12 Most likely high-molecular-weight multimers (HMWM) of von Willebrand factor (VWF) are damaged due to mechanical stress inside the ECMO circuit. 13 Increased cleavage by a disintegrin and metalloproteinase with a thrombospondin type 1 motif, member 13 (ADAMTS13) and binding of the VWF to platelets might also play a role in the development of aVWD. 14 Weaning from ECMO therapy can restore VWF function. 15 However, it can only be performed when respiratory function has stabilized. Therefore, best treatment strategy for aVWD in bleeding ECMO patients remains challenging.

## Methods

VWF-antigen (VWF:Ag), VWF-collagen-binding-activity (VWF:CB), and VWF-ristocetin-induced-binding (VWF:GP1bR) were determined applying HemosIL AcuStar-Assays (Instrumentation Laboratory, Werfen, Germany) including the calculation of VWF:GP1bR/Ag and VWF:CB/Ag ratio. VWF HMWM were analyzed using the Hydragel 5 von Willebrand Multimer kit and a Hydrasis 2 analyzer (both Sebia Labordiagnostische Systeme GmbH, Fulda, Germany).

## Case Description

**Fig. 1:**
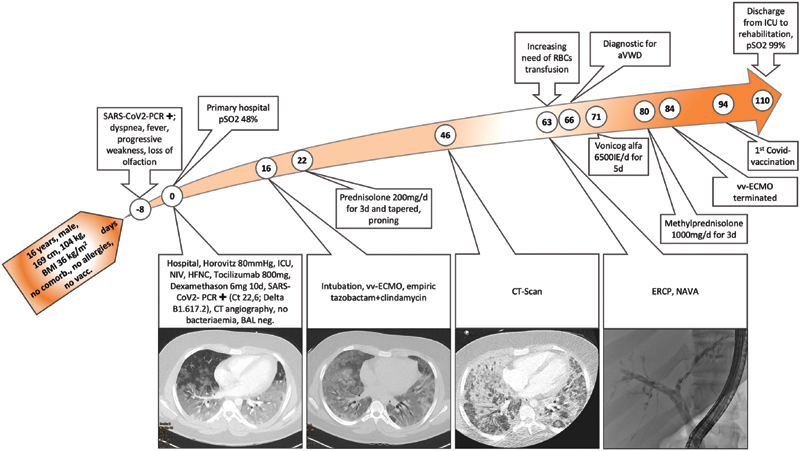
Presentation of patient's time course of disease since hospital admission (day 0). ICU, intensive care unit; NIV, noninvasive ventilation; HFNC, high-flow nasal cannula; PCR, polymerase chain reaction; BAL, bronchial lavage sampling; vv-ECMO, veno-venous extracorporeal membrane oxygenation; RBC, red blood cell transfusion; ERCP, endoscopic retrograde cholangiopancreatography; NAVA, neurally adjusted ventilator assist; pSO _2_ , partial oxygen saturation.

**Fig 2:**
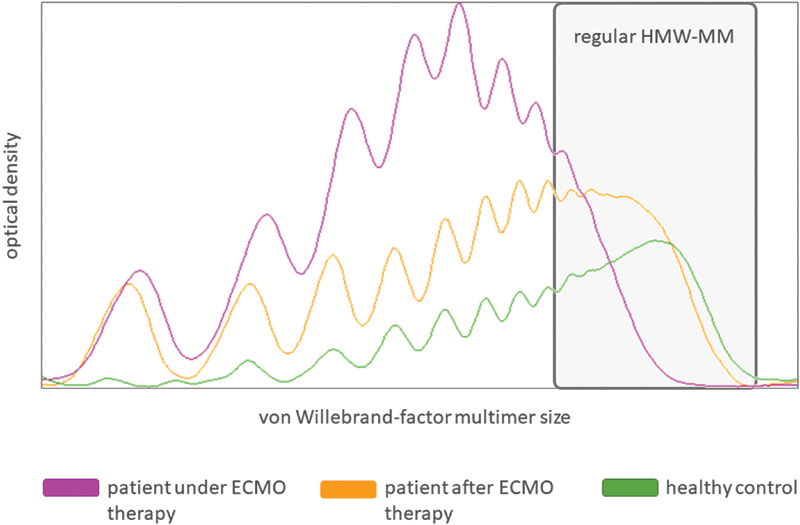
von Willebrand factor (VWF) multimer distributions on day 3 after bleeding occurred under and after ECMO therapy compared to healthy control. ECMO, extracorporeal membrane oxygenation; HMWMM, high molecular weight multimers.

A 16-year-old male patient was admitted to the intensive care unit (ICU) with severe dyspnea and hypoxia (SpO _2_ 48%) 1 week after reversed phase polymerase chain reaction confirmed SARS-COV-2 infection. Initially, the patient could be stabilized with nasal high-flow therapy and noninvasive ventilation. Sixteen days after ICU admission, the patient had to be intubated, but gas exchange remained persistently insufficient. Due to refractory hypoxemia, therapy with ECMO, cortisone, and prone positioning were initiated and adequate gas exchange could be restored immediately ( Fig. 1 ). Thoracic computed tomography showed severe bilateral milk glass opacities and increasing consolidation of pulmonary tissues. A minor peripheral pulmonary embolism was also detected. No venous thromboembolism could be diagnosed using repeated complete compression ultrasound of both lower limbs. The patient suffered from bacterial superinfection, recurrent sepsis and septic shock accompanied by liver dysfunction. Forty-seven days after the start of ECMO therapy, endoscopic papillotomy for hyperbilirubinemia due to sclerosing cholangitis had to be performed (endoscopic retrograde cholangiopancreatography [ERCP] picture is shown in right part of Fig. 1 ). As side effect, diffuse bleeding symptoms from the GI tract occurred, prompting the need for daily transfusion of 2 to 3 packs of red blood cell concentrate ( Supplementary Fig. S1 ). Repeated endoscopic attempts to achieve GI hemostasis failed. Intravenous unfractionated heparin therapy was stopped immediately and subsequently anti-Xa level decreased to < 0.1 IE/ml. Conventional laboratory coagulation parameters did not indicate any specific bleeding disorder ( Table 1 ). On the contrary, the patient exhibited highly elevated factor VIII levels, what is common in acute phase reaction. The VWF analysis was consistent with severe aVWD showing low ratios for VWF:CB/VWF:Ag (0.48) and VWF:GP1bR/VWF:Ag (0.5). Multimer analysis confirmed the diagnosis with abnormal distribution and nearly complete absence of HMWM compared to healthy controls ( Fig. 2 ). The patient was treated with recombinant human VWF (vonicog alfa), at a dose of 65 IE/kg daily for five consecutive days. Additionally, 2 g tranexamic acid were administered through gastral tube (indicated for enteral nutrition). After a single dose of recombinant VWF (rVWF), bleeding symptoms improved and the need for blood transfusion decreased ( Supplementary Fig. S1 ). Following the last dose, GI bleeding stopped and no further blood transfusions were necessary for several days. VWF:CB/VWF:Ag ratio increased during therapy with vonicog alfa to 0.64 and to 0.82 for VWF:GP1bR/VWF:Ag, respectively. Liver function recovered and the patient could be sufficiently weaned from ECMO therapy after 72 days. Multimer analysis 1 week after ECMO decannulation showed an adequate reappearance of HMWM ( Fig. 2 ). On day 110 the patient could be discharged from the ICU to a rehabilitation facility in stable condition ( Fig. 1 ).

**Table 1:** Laboratory parameters for bleeding course

	Day 1	Day 2	Day 3	Day 4	Day 5	Day 6	Day 7	Day 8	Day 9	Day 10	Day 11	Day 12	Day 13	Day 14	Reference range
Hemoglobin [mmol/L]	5.1	5.4	5.5	5.5	4.9	5.4	5.4	6.2	5.6	5.4	5.7	5.7	5.6	5.6	8.60 - 12.10
Erythrocytes [TPt/L]	2.71	2.95	2.97	2.95	2.77	2.92	2.90	3.25	2.97	2.76	2.99	2.95	2.92	2.95	4.6 - 6.2
Leucocytes [GPt/L]	8.39	9.43	10.76	11.51	15.32	14.13	11.64	12.02	10.80	11.39	12.29	10.67	10.16	5.87	3.8 - 9.8
Platelets [GPt/L]	114	64	56	49	88	96	91	78	91	120	154	132	164	127	150 - 400
INR	1.20	1.44	1.41	1.31	1.21	1.22	1.10	1.09	1.09	1.09	1.12	1.17	1.10	1.06	0.9 - 1.2
aPTT [s]	31	48	47	50	33	40	33	33	35	34	35	34	38	36	24 - 36
Factor VIII [%]	n.a.	n.a.	n.a.	> 400	n.a.	> 400	n.a.	> 400	n.a.	n.a.	> 400	> 400	n.a.	> 400	60 - 150
Fibrinogen [g/L]	6.72	9.77	10.33	9.67	9.35	6.53	6.25	6.58	6.33	6.31	6.07	6.00	5.83	5.35	2.0 - 4.0
D-dimers [ng/mL]	14033	7836	8984	7383	7714	6326	6886	7111	5723	4887	2962	2601	1898	1452	< 501
Anti-Xa activity [IU/ml]	< 0.10	0.11	< 0.10	0.13	< 0.10	< 0.10	n.a.	n.a	n.a	n.a.	n.a.	n.a.	n.a.	n.a.	0.1 to 0.3 a
VWF:CB (%)				> 191											61-193
VWF:Ag (%)				> 400											60-211
ratio VWF:CB/VWF:Ag				0.48											> 0.7
CRP [mg/L]	120.3	425.6	476.2	340.2	163.1	110.5	104.8	124.4	146.5	195.2	166.2	137.1	117.3	74.2	< 5.0
Procalcitonin [ng/mL]	6.83	8.84	1.68	0.87	0.69	0.61	0.40	0.37	0.30	0.23	0.30	0.41	0.39	0.37	< 0.50
Interleukin-6 [pg/mL]	n.a.	1359.0	267.0	115.0	n.a.	152.00	124.0	148.0	163.0	n.a.	95.1	91.2	50.5	54.1	< 7.0
Creatinine [µmol/L]	49	51	51	53	102	44	55	51	53	49	54	54	56	46	62 - 106
Bilirubin total [µmol/L]	327.4	377.4	452.5	558.4	545.3	591.6	682.4	756.8	753.6	751.5	733.6	703.4	688.8	647.6	< 21.0
Albumin [g/L]	21.8	24.0	22.4	21.9	19.1	23.4	25.2	26.1	26.8	27.6	26.8	25.9	28.0	26.6	35 - 52
SGPT [µmol/s*L]	2.18	1.87	1.55	1.56	1.49	1.25	1.30	1.40	1.35	1.59	1.96	2.65	3.24	3.31	< 0.75
SGOT [µmol/s*L]	3.47	3.12	2.63	3.04	3.53	3.04	3.32	3.07	2.85	3.10	3.98	5.37	6.61	6.55	< 0.77

Abbreviations: aPTT, activated partial thromboplastin time; CRP, C-reactive protein; ECMO, extracorporeal membrane oxygenation; INR, international normalized ration; n.a., not available; SGOT, serum glutamic-oxalacetic transaminase; SGPT, serum glutamic-pyruvic transaminase; UFH, unfractionated heparin; VWF:Ag, von Willebrand factor:antigen; VWF:CB, von Willebrand factor:collagen-binding-activity.

a There are no general recommendations available for target ranges of anti-Xa activity with UFH in ECMO patients.

## Discussion

While severe courses of COVID-19 where often seen in adults it is quite rare in children. A U.S. cohort study showed that of 167,262 children tested positive for SARS-CoV-2 10,245 (6.1%) were hospitalized, 1,423 (0.85%) met criteria for severe disease, and 42 (0.03%) required ECMO. 16 As in adults, obesity is associated with a severe course of COVID-19 in children, a condition with also was present in our case. 17 There is a growing body of evidence, that immune-related aspects might determine the development of severe causes. Therefore, immunomodulatory and anti-inflammatory therapies are recommended, especially in the early phase of COVID-19 disease. Whether a dysregulated immune response is also crucial for the failure in pulmonary improvement in the subsequent course remains hypothetical. However, it is known from other etiologies (viral infections, drugs, aspiration, rheumatological diseases) that the immune response can contribute to the development of a secondary organizing pneumonia (OP), a subtype of interstitial lung diseases. 18 For this reason, a high-dose prednisolone therapy can be indicated if OP is suspected. 19 Usually, an initial does of 0.75 to 1.5 mg/kg bodyweight (BW) of prednisolone for 4 weeks is used. 18 Considering secondary OP in an early phase of the COVID-19 disease is an established approach in our institution. We usually start with a dose of 2 mg/kg BW of intravenous prednisolone for 3 days, followed by 0.75 mg/kg BW for 2 weeks followed by subsequent tapering. As described above, this early therapy did not improve the condition of the patient. In severe cases of OP a high dose of intravenous methylprednisolone (500–1,000 mg) for 3 days is described. 19 This approach led to a substantial improvement of the respiratory system in our case.

Secondary sclerosing cholangitis (SSC) of the critical ill patient including COVID-19-related cholangiopathy (SC-CIP) is an underestimated and underdiagnosed disease with poor prognosis (1-year survival 55% without transplantation). 20 The SC-CIP is one entity of SSC, where toxic (drug associated) or hereditary forms are only few other examples of SSC types. The pathophysiology of SC-CIP is not well understood, but hypoxia of the biliary system seems to be a crucial factor leading to subsequent bile cast formation (composed of collagen from necrotic bile ducts 21 ) with superimposed biliary infections. 6 Due to commonly seen prolonged hypoxemic episodes in severe COVID-19 patients, the SC-CIP is a common complication in these patients affecting both, acute care 22 and long-term outcomes. 23 24 During critical care ursodeoxycholic acid, ERCP with sphincterotomy, evacuation of the sludge from the biliary system, as well as microbiological analyses of the bile with consequent anti-infective therapy for at least 2 weeks are recommended, although today there are no data available that show a stop of progression of SC-CIP due to this therapy. 6 Sphincterotomy led to an ongoing intraluminal bleeding in our case, what eventually led to the diagnosis of aVWD. aVWD is common in ECMO patients. Panholzer et al found that bleeding symptoms in ECMO patients occurred in 23% in combination with aVWD. 11 Start of the ECMO therapy induces changes in the coagulation system including low platelets, platelet function disorders, consumption of coagulation factors, and aVWD. However, these laboratory changes do not lead to clinically overt bleeding symptoms in all patients. Against this background, preemptive screening for coagulations disorders during ECMO therapy is useful, 25 especially before surgery. Few data are available on the prevalence of aVWD patients and its optimal treatment. Biguzzi et al presented a series of aVWD patients with GI bleeding and differing therapeutic approaches, recommending individualized treatment and control of underlying disease. 26 Kalbhenn and Zieger suggested an algorithm for prevention and therapy of aVWD-related bleeding in ECMO patients. 25 They emphasize the importance of bleeding prevention and recommend routine screening for coagulation disorder in ECMO patients. Although desmopressin is part of their suggested therapy concept, other studies showed that desmopressin may be less effective for treatment for aVWD. 2 The upregulation of factor VIII and VWF due to severe inflammation of the endothelium might be a reason for the reduced efficiency of desmopressin in these patients. Something similar had to be expected in our case. As shown in Table 1 the patient exhibited very high factor VIII levels, consistent with an inflammatory state. In this situation the ability of a further release of VWF and factor VIII from the endothelium by desmopressin is uncertain. Therefore, a VWF-containing product can be discussed in that situation. However, the elevated factor VIII levels should be taken into account when initiating therapy. COVID-19 acute respiratory distress syndrome is known for its coagulopathy with an increased risk for thromboembolic complications. 27 This, together with an ECMO circuit running in a patient should always be taken into consideration when giving coagulation factors because it can lead to both thrombosis in the patient and clotting in the ECMO circuit. Probably due to the shear stress within the ECMO circuit an unfolding of HMWM of VWF occurs. This makes them vulnerable to the cleavage by ADAMTS13 resulting in reduced HMWM levels. The simple evaluation of VWF:Ag or VWF:CB values without assessing the ratios could lead to the diagnosis being overlooked. This would have happened in the presented case. The confirmation of suspected VWD requires gel electrophoresis which is laborious and usually takes time. However, the ratio of VWF:CB/VWF:Ag is a rapid and reliable way to detect aVWD. The only causal treatment for ECMO-associated aVWD (i.e., the loss of high molecular multimers of VWF due to unfolding of VWF by the ECMO) is termination of the ECMO therapy, which requires sufficient pulmonary gas exchange. Therapeutic options for bleeding aVWD patients include plasma-derived concentrates containing factor VIII and/or VWF and rVWF concentrate (vonicog alfa) as well as adjuvant therapies such as tranexamic acid and desmopressin. 28 Therapeutic efficacy might be limited due to short half-life of the given VWF within the ECMO circuit and therefore the dose of rVWF has to be increased up to 65 IE/kg. Moreover, complex coagulopathy and hypercoagulability with increased factor VIII are a hallmark of severe COVID-19 disease, 29 leading to high number of thromboembolic events. 30 This may explain the pulmonary embolism in our patient, who exhibited elevated factor VIII levels ( Table 1 ), what also can disturb the determination of the activated partial thromboplastin time values. 31 rVWF, unlike plasma-derived VWF concentrates, does not contain factor VIII. This feature might be a particular advantage for patients with clinically relevant bleeding and simultaneous hypercoagulability due to severe COVID-19, sepsis, or similar critical conditions. Of note, the safe and effective use of rVWF for aVWD in pediatric patients as shown in the reported case is still off-label.

This case features the importance of aVWD in ECMO patients, the efficient use of rVWF concentrate in aVWD, and the high-dose methylprednisolone therapy in OP in a pediatric COVID-19 patient. Transfusion of rVWF should therefore be considered as a potential therapeutic strategy for ECMO patients bleeding due to aVWD and high levels of factor VIII at the same time. If OP is suspected a high-dose methylprednisolone therapy should be considered even after initial failure of prednisolone therapy.
