# Supplementary material for: Treatment of Acquired von Willebrand Disease due to Extracorporeal Membrane Oxygenation in a Pediatric COVID-19 Patient with Vonicog Alfa: A Case Report and Literature Review
Source: TH Open. 2023 Feb 23;7(1):e76–81. doi: 10.1055/a-2008-4367 (PMC9949976; doi:10.1055/a-2008-4367)
Supplement: Supplementary file 1 — Supplementary Material [file 10-1055-a-2008-4367-s22090040.pdf]

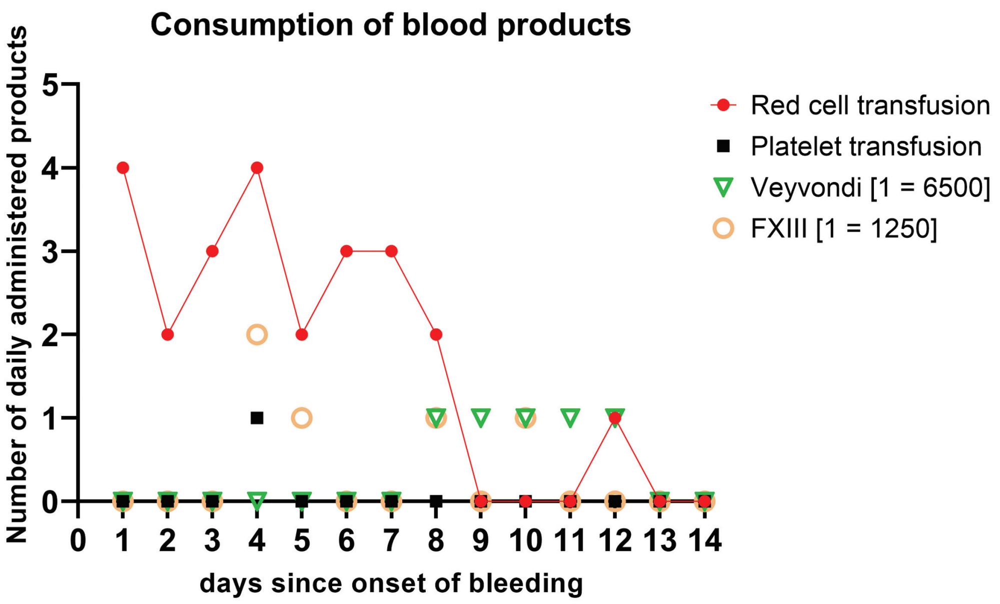

Supplementary Fig. S1 Time course of blood and coagulation product substitution. This figure shows the consumption of blood and blood product transfusion since onset of bleeding (day 63 since hospital admission).

 © 2023. The Author(s).
